# Serum Hepatocyte Growth Factor Concentration Correlates with Albuminuria in Individuals with Optimal Blood Pressure and Untreated Arterial Hypertension †

**DOI:** 10.3390/biomedicines12102233

**Published:** 2024-09-30

**Authors:** Margareta Fistrek Prlic, Ivana Vukovic Brinar, Jelena Kos, Zivka Dika, Ema Ivandic, Mirjana Fucek, Bojan Jelakovic

**Affiliations:** 1 Department of Nephrology, Arterial Hypertension, Dialysis and Transplantation, University Hospital Center Zagreb, 10000 Zagreb, Croatia; jkos980@yahoo.com (J.K.); ema_ivandic@yahoo.com (E.I.); 2 Department of Nephrology, Arterial Hypertension, Dialysis and Transplantation, University Hospital Center Zagreb, School of Medicine, University of Zagreb, 10000 Zagreb, Croatia; ivemedex@yahoo.com (I.V.B.); zivkadika1@gmail.com (Z.D.); jelakovicbojan@gmail.com (B.J.); 3 Department of Laboratory Diagnostics, University Hospital Center Zagreb, 10000 Zagreb, Croatia; mirjana.fucek@kbc-zagreb.hr

**Keywords:** hepatocyte growth factor, albuminuria, kidney injury, chronic kidney disease, arterial hypertension

## Abstract

**Background/Objectives:** Hepatocyte growth factor (HGF) is a protective factor against acute renal injury and chronic renal fibrosis. A positive correlation between HGF and blood pressure (BP) has been established. This study aimed to determine the association between serum HGF concentration and albuminuria in subjects with optimal blood pressure (OBP) and untreated arterial hypertension (UAH), as well as its association with BP levels, serum glucose levels, and inflammatory markers. **Methods:** Data from 563 subjects were analyzed. Albuminuria was normalized to urine creatinine and expressed as the albumin/creatinine ratio (ACR). HGF, serum glucose, C-reactive protein, and blood leucocyte counts were measured. BP was measured and subjects were divided into optimal blood pressure (BP < 120/80 mmHg, N = 295) and untreated arterial hypertension (BP > 140/90 mmHg, N = 268) groups. **Results:** The subjects with UAH were significantly older and had higher values of body mass index, waist circumference, serum total and LDL cholesterol levels, triglyceride levels, fasting glucose levels, and ACR (all *p* < 0.001). A significant positive correlation was found between serum HGF concentration and ACR in both groups. There was no difference or correlation between HGF and BP or inflammatory markers in either group. The multivariate regression analysis identified serum HGF concentration as a strong predictor of ACR increase (Beta = 0.376, *p* < 0.001). **Conclusion:** This study found that serum HGF concentration is associated with albuminuria not only in individuals with untreated arterial hypertension, but also in those with optimal blood pressure. The results suggest that serum HGF is an independent predictor of ACR increase in both groups.

## 1. Introduction

The rise in chronic kidney disease (CKD) worldwide can be attributed to the increased prevalence of conditions such as diabetes, arterial hypertension (AH), and hyperlipidemia, all of which can cause kidney damage. According to KDIGO guidelines, CKD is characterized by abnormalities in kidney structure or function that persist for at least 3 months and have implications for health [1,2]. CKD is classified based on its cause, glomerular filtration rate (GFR) category, and albuminuria category [1,2]. All these components are important for evaluating patients with CKD, and determining the cause of CKD is equally crucial. Screening for albuminuria is an important tool for detecting individuals with CKD. The early identification of CKD in at-risk individuals, who are often asymptomatic, can lead to better treatment options and prognosis. The preferred initial test for detecting albuminuria is the albumin/creatinine ratio. Elevated albuminuria (30 to 300 mg/24 h urine or ACR > 30 mg/g) is an early indicator of progressive kidney disease [1,2]. End-stage CKD is diagnosed based on the presence of renal fibrosis features, such as glomerulosclerosis, atherosclerosis, and tubulointerstitial fibrosis. These processes involve the progressive loss of renal cells and excessive deposition of extracellular matrix proteins. The prognosis for CKD patients is associated with the severity and extent of fibrotic lesions [3]. Hence, there is a need for regeneration-based therapies to prevent and potentially reverse the progression of renal fibrosis in CKD patients. Researchers have been investigating both clinical risk factors for CKD (e.g., arterial hypertension and diabetes) and the molecular mechanisms underlying renal injury and fibrosis. The highest-priority conditions for CKD detection are AH and diabetes [1,4,5]. For diabetes, the KDIGO guidelines recommend annual screening of people with diabetes for CKD. CKD screening should start at diagnosis of type II diabetes, since CKD is often already apparent at this time. For type I diabetes, screening is recommended 5 years after diagnosis [1]. AH is an independent risk factor for cardiovascular disease, and the epidemiological link between blood pressure (BP) and cardiovascular risk begins with very low blood pressure values (i.e., systolic BP 115 mmHg). The relationship between BP levels and cardiovascular and renal diseases is ongoing, and the line between normotension and AH is somewhat arbitrary [6]. AH is defined as the BP value at which the advantages of treatment (pharmacological or non-pharmacological) clearly outweigh the risks of treatment, as demonstrated by clinical trials [6,7]. AH is known to cause target organ damage (TOD), and this implies structural or functional changes in arteries or organs, including the heart, brain, and eyes, as well as the kidneys. The damage to the target organs is a marker of developed preclinical or asymptomatic cardiovascular disease [6]. Target organ damage is common in more severe or long-lasting AH. With more frequent and available diagnostics, TOD is diagnosed earlier in hypertensive patients [6]. If the patient has TOD, there is a greater cardiovascular risk, which becomes even greater if there is damage to multiple organs. If detected early, TOD can be positively influenced by antihypertensive therapy and changes in lifestyle habits, but in long-term AH, TOD cannot be improved despite better regulation of BP [6].

Hepatocyte growth factor (HGF) is a pleiotropic factor originating from the mesenchyme that regulates the growth, motility, and morphogenesis of different cell types, and it is therefore considered a humoral mediator in the interaction between the epithelium and the mesenchyme [8]. It is a polypeptide, heterodimeric glycoprotein that binds heparin and consists of two chains (69 kDa and 34 kDa) linked by a disulfide bond. HGF regulates diverse cellular processes such as cell survival, proliferation, migration, and differentiation [9,10]. The biological effects of HGF are mediated by the c-met receptor. HGF binding triggers c-met receptor activation by tyrosine autophosphorylation. This results in the recruitment of intracellular signaling molecules and the initiation of signal transduction cascades. HGF stimulates endothelial cell growth, stops cell apoptosis, and reduces cell death [11].

Previous data showed that the administration of exogenous cytokines, including HGF, have protective effects on injured kidneys [9,10,11]. Initially discovered to be a strong mitogen for mature hepatocytes, HGF was subsequently found to have similar effects on renal tubular cells [12]. In the kidneys, HGF is predominantly secreted by epithelial cells, mesangial cells, endothelial cells, and macrophages, while c-met receptors are located on fibroblasts and tubular epithelial cells [13,14]. In patients with acute kidney injury that require renal replacement therapies in the intensive care units (ICUs), the decreased levels of urinary HGF in the first 14 days are associated with an increased probability of renal recovery [15]. HGF speeds up renal tubular repair, resulting in a quick recovery of tubular morphology and function [16]. In a mouse model of chronic renal disease, exogenous HGF administration prevented renal dysfunction and chronic mesangial damage and had therapeutic benefits for end-stage renal disease [17]. Moreover, HGF might contribute to renal vasculature protection in injured kidneys lacking angiogenic factors [18,19]. HGF also improves blood flow via nitric-oxide-synthase-mediated vasodilation [19]. These findings suggest that HGF suppresses fibrosis in addition to maintaining blood flow in injured kidneys. In vitro studies have demonstrated that the balance between TGF-β1 and HGF seems to play a critical role in determining whether the injured tissues undergo recovery or fibrogenesis. The addition of recombinant human HGF to renal fibroblast culture media dose-dependently inhibited TGF-β1 mRNA expression and reduced collagen III secretion [20]. Therefore, HGF and c-met are potential targets for the treatment of kidney diseases. ANG-3777, produced by Angion Biomedica Corporation, is a small molecule with HGF-like activity [21]. In phase 2 and 3 studies on renal transplantation patients showing signs of delayed graft function, improved renal function in subjects treated with ANG-3777 is observed, with a good safety profile [22,23].

Previous studies have also shown that serum HGF concentration is significantly correlated with systolic BP and is higher in hypertensive individuals compared to normotensive individuals [24]. In hypertensive individuals, serum HGF secretion increases in response to high BP to prevent endothelial dysfunction [24,25]. Additionally, the concentration of HGF in the serum of hypertensive patients with target organ damage is significantly higher than in those without complications [25]. Higher concentrations of HGF have also been observed in patients with type 2 diabetes, especially those with AH [26]. The concentration of serum HGF in subjects with type 2 diabetes was significantly higher than in healthy control subjects, even when the subjects with diabetes did not have AH [26]. It has also been proven that people with type 2 diabetes have particularly elevated HGF values when atherosclerotic changes are present, regardless of BP values [25,26]. Moreover, patients with type 2 diabetes have higher concentrations of HGF when they have diabetic retinopathy, and the measurement of serum HGF could be useful in predicting the presence of diabetes complications [26]. Overall, HGF is a potent antifibrotic protein that inhibits kidney fibrosis through several mechanisms. Thus, the HGF/c-met signaling pathway plays an important role in protecting podocytes from injury, thereby reducing proteinuria and albuminuria [27,28]. Previous studies have demonstrated the positive correlation between HGF concentration and inflammatory markers, namely C-reactive protein, in patients with CKD and liver disease [29,30]. Higher concentrations of serum HGF predicted worse survival outcomes in CKD patients requiring dialysis [30].

We aimed to determine the association between serum HGF concentration and albuminuria in subjects with optimal blood pressure and subjects with untreated arterial hypertension. Additionally, we analyzed the association between HGF concentrations and BP levels, as well as serum glucose levels and inflammatory markers in both groups.

## 2. Materials and Methods

We conducted a clinical and epidemiological study in villages in eastern Croatia. We selected adult residents with a glomerular filtration rate (GFR) greater than 60 mL/min/1.73 m^2^. Inclusion criteria were age over 18 years and no known history of treated arterial hypertension. The subjects were not using other medications (such as non-steroidal anti-inflammatory drugs, corticosteroids, COX2 inhibitors, or statins) that could have affected the laboratory analyses. Exclusion criteria included pregnancy, breastfeeding, active malignant disease, acute illness, and a personal history of inflammatory disease. The study was approved by the School of Medicine University of Zagreb Ethical Committee.

After applying the exclusion criteria, the subjects were divided into groups based on their blood pressure (BP): those with optimal blood pressure (BP < 120/80 mmHg) and those with untreated arterial hypertension (UAH, BP > 140/90 mmHg). All subjects were informed about the research, its objectives, benefits, and potential risks, and signed an informed consent to participate in the study.

All subjects underwent a clinical examination, and their body height, body mass, and waist circumference were measured. The body mass index (BMI) was calculated using the standard formula (BMI = body weight/height^2^). Blood pressure was measured with an oscillometric sphygmomanometer while sitting after 5 min of rest. BP was measured on two occasions for accuracy, resulting in a total of 6 BP measurements. Fasting blood samples and morning urine samples were collected. All laboratory tests were performed using standard routine laboratory procedures immediately after arrival in the laboratory. The laboratory analysis was carried out at the Department of Laboratory Diagnostics of the Clinical Hospital Center Zagreb and the Faculty of Medicine of the University of Zagreb. The complete blood count was determined according to the principle of laser light scattering technology (Sysmex XE 5000 device, Kobe, Japan). Serum creatinine levels were determined from centrifuged blood (10 min at 3500 revolutions at room temperature) by Jaffe’s kinetic method within the concentration range 18–2200 μmol/L (Olympus AU 2700 analyzer, Tokyo, Japan, reagent Beckman-Coulter, Brea, CA, USA). Serum glucose levels were determined by UV photometry with hexokinase, triglycerides by photometry with glycerol phosphate oxidase (GPOPAP), total cholesterol by photometry with cholesterol oxidase, HDL cholesterol by homogeneous enzyme-34 immunoinhibition method, and LDL cholesterol by homogeneous method with CHE and CHO. Creatinine in urine was analyzed by the same method as serum creatinine concentration (device Olympus AU 2700 analyzer, reagent Beckman-Coulter) with automatic dilution of urine and normal saline in 1:10 ratio. Urine albumin was measured in the first morning sample by the latex immunonephelometric method with standardized application of the primary calibrator, ERM-DA470, on the Behring device Nephelometer Analyzer II (King of Prussia, PA, USA) with method sensitivity of 3.0 mg/L. C-reactive protein was measured by two-reagent immunoturbidimetric system. Measurement of serum HGF concentration was performed using ELISA enzyme immunoassay in Brigham and Women’s Hospital in Boston, USA. Albuminuria was expressed as albumin-to-urine ratio (mg/g). The glomerular filtration rate was determined based on the Cockroft–Gault formula (GFR = {(140 − age in years) × body mass in kg/creatinine(s) × 72 × (0.85 in women)). All data were entered into an electronic database and statistically processed. Numerical variables were checked for normality of distribution using the Kolmogorov–Smirnov test. Non-parametric tests (Mann–Whitney U or Kruskal–Wallis) were used to test differences between groups of numerical and ordinal variables. To test the difference between groups of nominal categorical variables, either χ2 or Fisher’s exact test was used. The correlation of numerical variables was tested with the Spearman Rho test. The level of statistical significance was chosen at α = 0.05. Multivariate linear regression analysis was used to analyze the association of variables. Variables that were considered to have a cause-and-effect relationship were included in the analysis. The forward stepwise method was used. ACR was set as dependent variables, and the association of ACR with age, gender, serum glucose concentration, systolic blood pressure, and HGF was analyzed. The program package STATA was used for data processing (STATA/MP ver. 15.1, StataCorp LLC, College Station, TX, USA).

## 3. Results

Overall, 563 subjects (57.8% women) were enrolled in the study, 295 in the OBP group and 268 subjects in the UAH group. The subjects with OBP were, on average 37.1, years old, and had a normal body mass index and an average BP of 110.5/71.9 mmHg. The average blood glucose, total cholesterol, blood triglycerides, and albuminuria values are listed in Table 1.

The subjects with untreated arterial hypertension were, on average, 58.5 years old and overweight (BMI 28.7 kg/m^2^). The average arterial pressure in this group was 157.3/91.9 mmHg. The average values of blood glucose, total cholesterol, blood triglycerides, and albuminuria (ACR) for UAH are listed in Table 2.

When compared to the OBP group, the subjects in the UAH group were significantly older and had higher values of body mass index, waist circumference, serum total and LDL cholesterol levels, triglyceride levels, and fasting glucose levels (all *p* < 0.001), as shown in Table 3. The subjects with UAH had significantly higher ACR values.

The serum HGF concentrations were also found to be higher in the subjects with untreated arterial hypertension, although the difference was not significant (Table 4).

A multivariate linear regression analysis was performed to analyze the relationship between the dependent variable, HGF, and the variables of age, gender, systolic and diastolic blood pressure, and serum glucose levels. It was found that age is an independent predictor of an increase in HGF, while gender, systolic and diastolic blood pressure, leucocyte count, serum C-reactive protein, and serum glucose levels are not predictors of HGF increase (Table 5).

It was also observed that the subjects with higher ACR values had higher serum HGF concentrations in both groups, but again, the difference was not significant (*p* = 0.747 for OBP an *p* = 0.066 for UAH, respectively). No significant correlation was found between the concentration of HGF and the values for systolic BP and diastolic BP in either group. Moreover, no correlation was found between HGF concentration and leukocyte and CRP values in either group (all *p* > 0.05). The data are presented in Table 6. However, a significant positive correlation was found between serum HGF concentration and ACR in both groups (Table 6).

The untreated hypertensives (UAHs) also had significantly higher serum glucose compared to the OBP group (*p* < 0.001). In the UAH group, a statistically significant positive correlation was found for serum glucose levels and HGF concentration (*p* = 0.04). However, no significant correlation between HGF and serum glucose levels was observed in the OBP group (*p* = 0.6). Moreover, 31 subjects in the UAH group (11.5%) fulfilled the criteria for diabetes (fasting blood glucose levels > 7 mmol/L) compared to 12 subjects in the OBP group (4%) [5].

A multivariate linear regression was performed on a sample of all the subjects to analyze the relationship between the dependent variable, ACR, and age, gender, serum glucose levels, systolic BP, and HGF (variables considered to have a cause-and-effect relationship were included). The analysis found that age, gender, eGFR, and glucose levels are not predictors of albuminuria increase (ACR), while HGF is an independent predictor of albuminuria increase (ACR). Systolic BP is also an independent predictor of albuminuria increase (ACR). The data are presented in Table 7.

## 4. Discussion

In our study, we conducted a prospective clinical and epidemiological investigation involving a large number of participants. We took multiple blood pressure measurements on two separate occasions, allowing for a more accurate classification of the individuals. The limitation of our extensive epidemiological study is that we were only able to take samples and determine laboratory parameters at one time.

Previous studies have shown that the concentration of serum HGF is significantly correlated with BP, predominantly systolic BP. The correlation is more significant in hypertensive than in normotensive individuals [24]. HGF is a pleiotropic factor originating from the mesenchyme that regulates the growth, motility, and morphogenesis of different types of cells, and it is therefore considered a humoral mediator in the interaction between the epithelium and the mesenchyme [8]. Hypothetically, serum HGF is increased in hypertensive individuals to prevent endothelial dysfunction [24,25]. Bussolino et al. also found that the serum concentration of HGF in hypertensive patients with developed complications in terms of target organ damage is higher than in hypertensive patients without organ damage [31]. In our study, the concentrations of HGF show an increasing trend, corresponding to the increase in BP. Despite this increasing trend in serum HGF concentration, there was no significant difference in concentrations between the OPB and UAH groups. We found no significant correlation between HGF and BP in our study.

In comparing the concentration of HGF with other inflammatory biomarkers, the correlation of the concentration between HGF and blood leukocytes, as well as CRP levels, was not verified. Some studies have shown a positive correlation between the serum value of HGF and CRP in patients with other acute or chronic conditions, e.g., chronic liver disease and chronic renal failure requiring renal replacement, or after acute myocardial infarction [29,30,32].

The untreated hypertensive patients had significantly higher serum glucose compared to the OBP group (*p* < 0.001). A statistically significant positive correlation was found between serum glucose values and HGF concentration only in the UAH group, and as previously stated, other studies also found higher HGF concentrations in diabetics, especially those with pronounced atherosclerotic changes and retinopathy [26].

Regarding subjects with untreated arterial hypertension, the ACR is significantly higher in this group compared to the OBP groups, which could indicate renal damage in our UAH subjects, a well-established complication of arterial hypertension [1]. We found a significant positive correlation between the concentration of HGF and albuminuria in both the OBP and UAH groups.

A multivariate linear regression analysis was performed, according to which systolic blood pressure and HGF are independent predictors of increasing ACR. Age, gender, and serum glucose levels were not independent predictors of ACR increase.

Studies conducted on animal models showed conflicting results on the association between HGF and albuminuria. The intravenous administration of recombinant HGF led to the appearance of albuminuria in mice, while other studies showed the protective role of HGF in podocyte function and the consequent reduction in albuminuria [27,28,33]. Therefore, we could hypothesize that an increase in serum HGF concentration is a response to podocyte injury and the subsequent increase in albuminuria. HGF and its biological effects mediated through the c-met receptor have a promising role in developing novel therapeutic agents for kidney injury. Several studies on small HGF-like molecule (ANG-3777) efficacy have already been conducted, showing a promising effect on delayed kidney graph function [22,23]. However, more studies are needed to confirm the potential role of this molecule in treating albuminuria.

As previous studies have shown, damage to the target organs is a marker of developed asymptomatic cardiovascular disease [6]. Target organ damage, including albuminuria, is common in severe or long-lasting AH; however, the early detection of this condition should be a priority in the clinical care of patients with AH. With more frequent and available diagnostics, including the routine measurement of ACR in urine, kidney damage is diagnosed earlier in hypertensive patients [6]. If kidney injury is detected early, it can be positively influenced by medicaments and changes in lifestyle habits [6].

## 5. Conclusions

This study found that serum HGF concentration is associated with albuminuria not only in individuals with untreated arterial hypertension but also in those with optimal blood pressure. The results suggested that serum HGF was an independent predictor of ACR increase in both groups. Regarding all the data on kidney damage and the damaging role of arterial hypertension, as well as the results of our study, a question could be raised as to whether serum HGF concentration should be used as a clinical biomarker of early kidney injury alongside the ACR ratio. Despite previous studies confirming a significant positive correlation of HGF and BP or inflammatory markers, we did not find such correlations in our study. More studies are needed to confirm the diagnostic and prognostic value of serum HGF concentration in several clinical conditions, including arterial hypertension, inflammatory diseases, and renal injury. Moreover, HGF and its biological effects mediated through the c-met receptor have a promising role in developing novel therapeutic agents for kidney injury.

## Figures and Tables

**Table 1:** Clinical characteristics and laboratory data of subjects with optimal blood pressure.

				Interquartile Range	
	$\overset{\prime }{x}$	SD	Med	25%	75%
**Age**	37.1	13.6	35.0	26.3	45.0
**Height (cm)**	168.9	9.7	168.0	162.0	175.0
**Body weight (kg)**	70.5	13.8	69.0	61.0	79.0
**Waist circumference (cm)**	85.6	12.0	85.0	77.0	94.0
**BMI (kg/m^2^)**	24.7	4.3	24.1	21.6	27.4
**Heart frequency (beats/min)**	77.0	12.8	76.0	68.5	85.5
**Systolic BP (mmHg)**	110.5	6.8	111.0	106.5	116.5
**Diastolic BP (mmHg)**	71.9	7.1	72.5	67.5	77.0
**Blood leucocytes (×10^9^/L)**	6.4	1.6	6.0	5.2	7.3
**Blood glucose (mmol/L)**	4.8	0.5	4.8	4.5	5.1
**C-reactive protein (mg/L)**	2.1	4.4	0.9	0.4	2.3
**Total cholesterol**	5.1	1.0	5.0	4.3	5.8
**HDL cholesterol**	1.5	0.3	1.5	1.3	1.7
**LDL cholesterol**	3.1	0.9	3.0	2.4	3.6
**Triglycerides**	1.2	0.8	1.0	0.7	1.4
**ACR mg/g**	14.3	111.1	4.0	3.0	6.3

ACR—albumin–creatinine ratio in urine, BMI—body mass index, SD—standard deviation, $\overset{\prime }{x}$—arithmetic mean, Med—median, BP—blood pressure.

**Table 2:** Clinical characteristics and laboratory data of subjects with untreated arterial hypertension.

				Interquartile Range	
	$\overset{\prime }{x}$	SD	Med	25%	75%
**Age**	58.5	14.6	59.0	47.8	70.0
**Height (cm)**	168.2	11.0	168.0	160.0	176.0
**Body weight (kg)**	81.1	17.1	80.0	70.0	91.0
**Waist circumference (cm)**	98.0	13.2	98.0	89.0	106.0
**BMI (kg/m^2^)**	28.7	5.8	28.1	24.7	31.7
**Heart frequency (beats/min)**	79.2	11.4	77.5	71.6	86.4
**Systolic BP (mmHg)**	157.3	15.6	154.0	145.5	166.0
**Diastolic BP (mmHg)**	91.9	11.2	92.5	84.5	99.0
**Blood leucocytes (×10^9^/L)**	6.6	1.7	6.3	5.4	7.4
**Blood glucose (mmol/L)**	5.7	1.6	5.5	5.0	6.0
**C-reactive protein (mg/L)**	3.6	7.5	1.6	0.9	3.4
**Total cholesterol (mmol/L)**	6.1	1.2	6.0	5.2	6.9
**HDL cholesterol (mmol/L)**	1.6	0.4	1.6	1.3	1.8
**LDL choelsterol (mmol/L)**	3.8	1.1	3.7	3.0	4.4
**Tryglicerides (mmol/L)**	1.7	1.5	1.4	1.0	2.0
**ACR mg/g**	27.5	102.1	5.8	3.5	13.9

ACR—albumin–creatinine ratio in urine, BMI—body mass index, SD—standard deviation, $\overset{\prime }{x}$—arithmetic mean, Med—median, BP—blood pressure.

**Table 3:** Comparison of clinical characteristics of both groups.

	Mann–Whitney U	*p* Value
**Age**	11,162	<0.001
**Height (cm)**	33,635	0.581
**Body mass (kg)**	21,173	<0.001
**Weight circumference (cm)**	15,402	<0.001
**BMI (kg/m^2^)**	19,425	<0.001
**Heart frequency (/min)**	35,433	0.101
**Systolic BP (mmHg)**	3234	<0.001
**Diastolic BP (mmHg)**	5219	<0.001
**Blood leucocytes (×10E^9^/L)**	20,790	0.308
**Blood glucose (mmol/L)**	9854	<0.001
**C-reactive protein (mg/L)**	6638	<0.001
**Total cholesterol (mmol/L)**	12,888	<0.001
**HDL cholesterol (mmol/L)**	20,958	0.142
**LDL cholesterol (mmol/L)**	13,792	<0.001
**Tryglicerides (mmol/L)**	14,072	<0.001
**ACR mg/g**	22,776	<0.001

**Table 4:** Serum HGF concentrations for both groups.

Group	Median Serum HGF Concentration (pg/mL)	Interquartile Range 25–75%	*p* Value
Optimal blood pressure (OBP)	270.8	153.7–456.5	0.078
Untreated arterial hypertension (UAH)	304.2	198.8–587.9	

HGF—hepatocyte growth factor, *p*—*p* value, level of significance.

**Table 5:** Multivariate regression analysis (dependent variable—HGF, independent variables—age, gender, serum glucose level, systolic BP, diastolic BP, and C-reactive protein).

Dependent Variable HGF	Beta	*p*
**Age**	0.258	0.001
**Gender**	−0.055	0.305
**Systolic BP**	−0.046	0.583
**Diastolic BP**	0.098	0.178
**Serum glucose level**	−0.002	0.975
**C-reactive protein**	−0.020	0.793

HGF—hepatocyte growth factor, BP—blood pressure, *p*—*p* value, level of significance.

**Table 6:** Correlation between serum HGF concentration, ACR, blood pressure, and inflammatory markers for both groups.

Group	ACR	Systolic BP	Diastolic BP	Leucocytes	CRP
**OBP**					
HGF rS	0.202	−0.037	0.072	−0.003	−0.022
*p*	0.020	0.671	0.413	0.972	0.839
**UAH**					
HGF rS	0.230	0.135	0.027	0.021	0.019
*p*	0.019	0.163	0.778	0.833	0.920

ACR—albumin–creatinine ratio in urine, HGF—hepatocyte growth factor, OBP—optimal blood pressure, UAH—untreated arterial hypertension, BP–blood pressure, CRP—C-reactive protein, rS–Spearman Rho correlation coefficient, *p*—*p* value, level of significance.

**Table 7:** Multivariate regression analysis (dependent variable—ACR, independent variables—age, gender, serum glucose, systolic BP, and HGF).

Dependent Variable—ACR	Beta	*p*
**Age**	0.038	0.492
**Gender**	0.016	0.689
**Serum glucose**	0.035	0.400
**SBP**	0.134	0.028
**HGF**	0.376	0.000

ACR—albumin-creatinine ratio in urine, HGF—hepatocyte growth factor, SBP—systolic blood pressure, *p*—*p* value, level of significance.

## Data Availability

All research data can be shared upon request.
